# Supplementary material for: Drought characteristics and their impact on vegetation net primary productivity in the climate-sensitive transition zones of North China
Source: PLoS One. 2026 Feb 25;21(2):e0343746. doi: 10.1371/journal.pone.0343746 (PMC12935246; doi:10.1371/journal.pone.0343746)
Supplement: S1 Table — (DOCX) [file pone.0343746.s001.docx]

**S1 Table** Changes in the area of dry and wet regions

|  | Humid zone(%) | Semi-humid zone(%) | Semi-arid zone(%) | Arid zone(%) |
| --- | --- | --- | --- | --- |
| 2001 | 0.04 | 8.84 | 88.68 | 2.44 |
| 2002 | 0.03 | 6.85 | 91.65 | 1.47 |
| 2003 | 10.46 | 44.97 | 44.17 | 0.40 |
| 2004 | 1.58 | 16.92 | 79.32 | 2.18 |
| 2005 | 3.53 | 28.11 | 64.55 | 3.81 |
| 2006 | 0.94 | 12.01 | 84.95 | 2.10 |
| 2007 | 3.28 | 25.48 | 70.25 | 1.00 |
| 2008 | 0.66 | 23.75 | 73.47 | 2.12 |
| 2009 | 0.11 | 16.20 | 81.33 | 2.35 |
| 2010 | 4.95 | 22.88 | 70.79 | 1.37 |
| 2011 | 1.14 | 26.51 | 71.53 | 0.83 |
| 2012 | 4.27 | 19.78 | 75.49 | 0.47 |
| 2013 | 3.01 | 14.78 | 80.76 | 1.45 |
| 2014 | 0.11 | 13.29 | 85.61 | 0.99 |
| 2015 | 0.16 | 16.83 | 81.21 | 1.81 |
| 2016 | 0.78 | 31.69 | 66.49 | 1.04 |
| 2017 | 0.11 | 15.66 | 83.47 | 0.76 |
| 2018 | 0.30 | 23.69 | 75.95 | 0.07 |
| 2019 | 0.46 | 11.61 | 86.37 | 1.56 |
| 2020 | 4.91 | 29.64 | 64.26 | 1.19 |
| Annual rate | -0.0008 | 0.00083 | 0.00069 | -0.00066 |
