# Supplementary figures and images for: Drought characteristics and their impact on vegetation net primary productivity in the climate-sensitive transition zones of North China
Source: PLoS One. 2026 Feb 25;21(2):e0343746. doi: 10.1371/journal.pone.0343746 (PMC12935246; doi:10.1371/journal.pone.0343746)

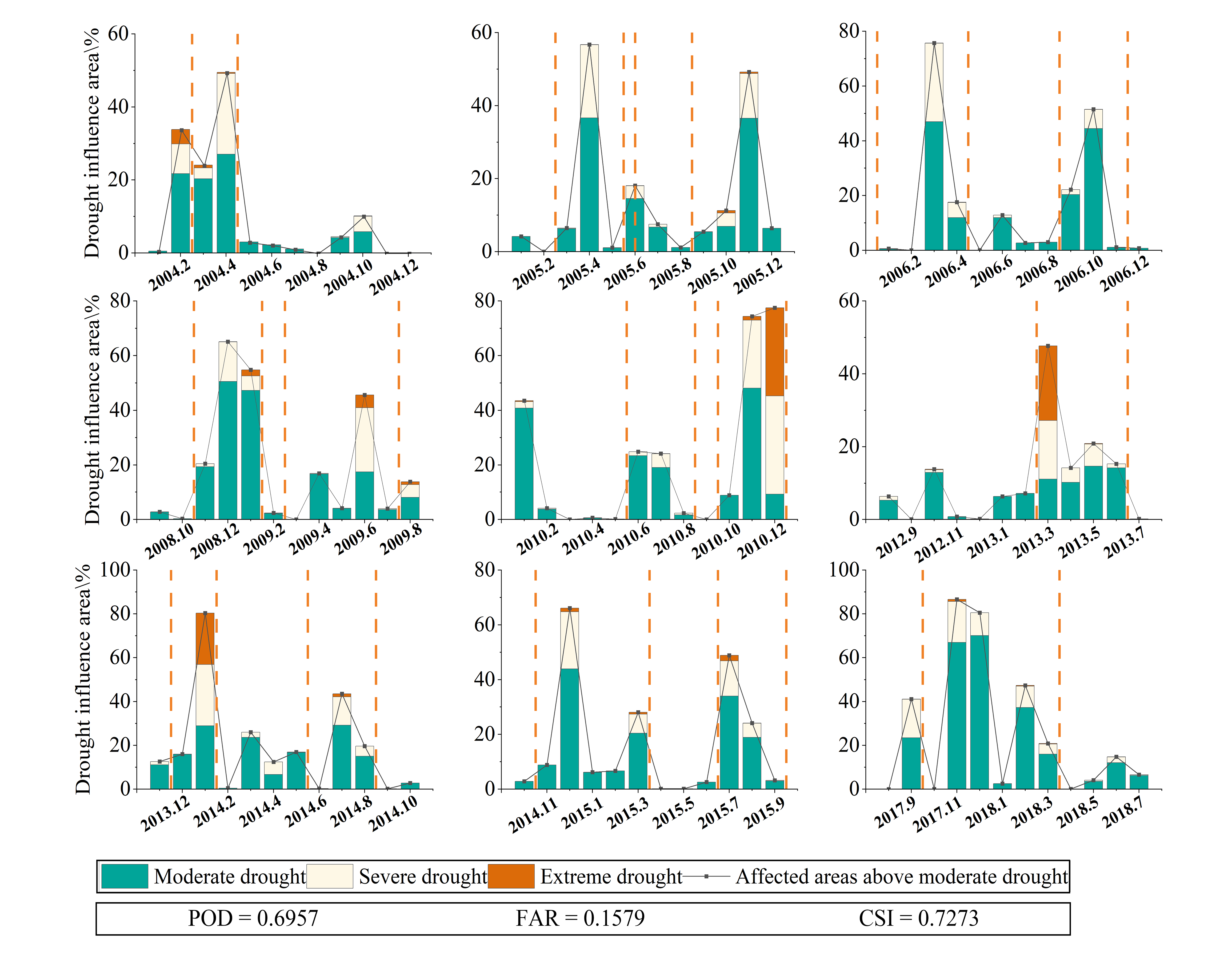

Supplement: S1 Fig — Additionally, calculate the Probability of Detection (POD), False Alarm Ratio (FAR), and Critical Success Index (CSI) for these historical drought events and the SPEI-based drought estimates, in order to assess the accuracy of the SPEI index. (The orange dashed lines indicate the historical drought periods: the spring drought in 2004; the spring and summer drought in 2005; the spring and autumn drought in 2006; the winter, spring, and summer drought from November 2008–2009; the summer and autumn drought in 2010; the spring drought in 2013; the winter, spring, and summer drought from December 2013–2014; the winter, spring, and summer drought from November 2014–2015; and the consecutive autumn and winter droughts from October 2017–2018.). (TIF) [file pone.0343746.s002.tif]

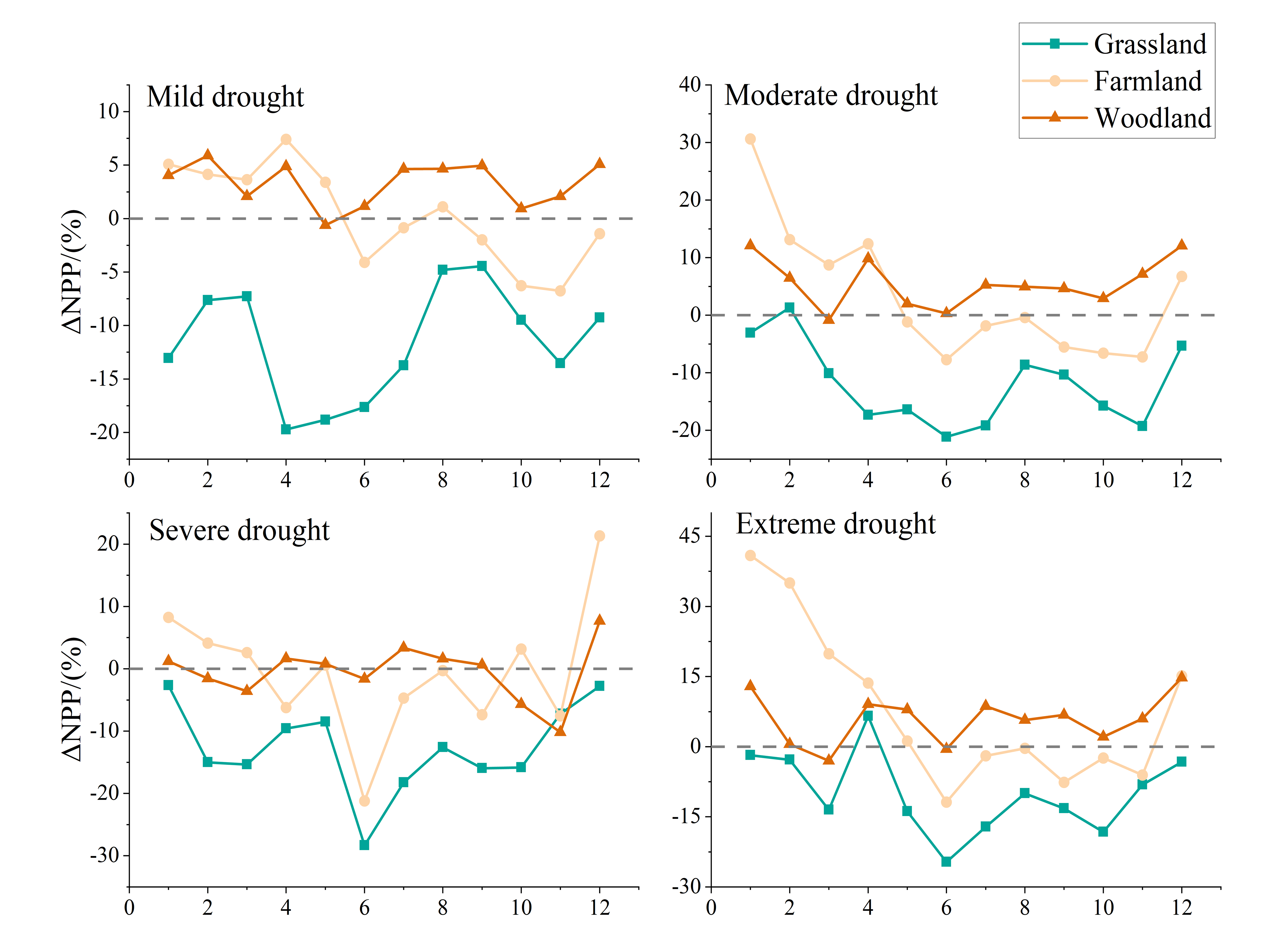

Supplement: S2 Fig — ΔNPP represents the percentage change in NPP relative to the baseline value, which is calculated using the average NPP of vegetation in months without drought across different years. (TIF) [file pone.0343746.s003.tif]
