# Supplementary material for: Drought characteristics and their impact on vegetation net primary productivity in the climate-sensitive transition zones of North China
Source: PLoS One. 2026 Feb 25;21(2):e0343746. doi: 10.1371/journal.pone.0343746 (PMC12935246; doi:10.1371/journal.pone.0343746)
Supplement: Attachment — Submitted filename: Response to Reviewers.docx [file pone.0343746.s005.docx]

**Response to the Reviewer's Comments**

**Submission ID:** PONE-D-25-53876

**Title:** Drought Characteristics and Their Impact on Vegetation Net Primary Productivity in the Climate-Sensitive Transition Zones of North China

**Comments from the Editor:**

**Response:** We thank all the editors and reviewers for their valuable comments and suggestions. We have carefully revised the manuscript to enhance its clarity and facilitate the understanding of the readers. Our point-to-point responses are presented below. We hope that the revision would satisfactorily address the comments and concerns of the editors and reviewers.

Regarding the journal requirements, the following modifications have been made:

1. I have ensured that my manuscript complies with the formatting requirements of PLOS ONE.
2. Funding information has been explicitly stated within the manuscript.
3. Firstly, the website addresses from which the data were obtained have been provided in the data description section of the submission system and can be accessed through these links. Secondly, the map of the study area was created using open-source software, based on the “Comprehensive Natural Geographic Division” established by Mr. Zhao Songqiao (relevant literature has been cited). The data website address is: “https://osgeo.cn/map/m0148/”. This data website is supported by the Open Source Geospatial Foundation, a non-profit organization whose main goal is to promote the collaborative development of open-source geospatial technologies and data. There are no copyright issues regarding these data. Additionally, in the manuscript, the relevant website addresses for the data sources (such as temperature, precipitation, solar radiation, SPEI datasets, NPP datasets, and land use type datasets) have been listed, and references have been provided. These data are publicly available, and their use in scientific research papers, with proper citation, is completely permissible and does not involve any copyright issues.In case the website may become inaccessible, the screenshot of the web page containing the map of the research area is provided below:

4. All other upload issues have been strictly addressed according to the requirements.

**Reviewer#1：**

**Response:** Thanks for the positive comments.

**Comment1：**The manuscript contains multiple instances of writing errors and the author must carefully review it. For example, in sections such as the abstract and keywords, "climate-sensitive" is incorrectly written as "limate-sensitive." Additionally, a multiplication sign (×) is missing in Equation (2), and there is a error in line 314 with "figd."

**Response:** Thank you for providing such detailed suggestions. I have carefully proofread and revised the manuscript, correcting errors in spelling, phrasing, and symbols.

**Comment2：** In the data processing methods section, the author used DFI to calculate drought frequency. The calculation formula for DFI should be provided, clarifying which drought index was used for the computation.

**Response:** The formula for the Drought Frequency Index (DFI) has been supplemented with additional explanations. Beyond formula revisions, the use of SPEI for drought identification has been clarified. The introduction section explicitly states that SPEI is primarily employed for overall drought identification, while SSMI is mainly used to identify drought through soil moisture. Therefore, SSMI is combined with SPEI to explore the impact of drought on vegetation NPP.

**Please see below for the details:**

“ The drought frequency index (DFI) measures the ratio of drought months to total months during the study period. Higher DFI values indicate greater drought frequency [35]. When SPEI ≤ -1, it is considered that a “drought” has occurred. The formula for calculating the frequency of droughts is given in Equation (3):

| $DFI=\left(\frac{m}{M}\right)\times100\%$ | (3) |
| --- | --- |

Where DFI represents the frequency of droughts; m denotes the number of months during which droughts occur; M represents the total number of months within this time scale. ”( Page 9, Lines 178 - 183 )

“ The Standardized Soil Moisture Index (SSMI) is an agricultural drought monitoring indicator constructed based on historical soil moisture time series. This method not only features a relatively straightforward calculation process but also accounts for the distributional characteristics of soil moisture data. Research on its applicability in regional agricultural drought monitoring provides a scientific basis for establishing operational drought monitoring systems and assessing their impacts [36]. SPEI can provide early warnings of climate risks associated with water deficits, while SSMI can verify whether drought has already caused substantive impacts on surface ecosystems and agricultural production. The two indices form a highly complementary relationship in terms of physical mechanisms, spatiotemporal attributes, and monitoring targets. The combined use of SPEI and SSMI overcomes the limitations of individual indices, providing a more systematic, multidimensional, and reliable scientific framework for drought early warning, precise assessment, process tracking, and impact analysis. Therefore, this study employs the SPEI index to analyze drought characteristics from 2001 to 2020, assess their impact on NPP, and utilize the SSMI to validate the substantive effects of drought on NPP in terrestrial ecosystems. ” ( Pages 3-4, Lines 59 - 72 )

**References：**Zhou, H.K., Wu, J.J., Li, X.H., et al. Suitability of assimilated data-based standardized soil moisture index for agricultural drought monitoring. Acta Ecol Sin. 2019;39:2191–2202. https://doi.org/10.5846/stxb201801190153.

**Comment3 :** The manuscript divides the drought class according to the SSMI index(Table 2), but a significant disconnect emerges in the subsequent core analysis: SSMI is only used to validate interannual trend consistency with SPEI-12. The manuscript fails to quantify the impacts of different SSMI drought levels on vegetation NPP based on：this classification standard. Furthermore, throughout the analysis of drought effects on NPP, only SPEI is employed as the drought indicator, with no incorporation of the SSMI classification into the evaluation. This renders the design of the SSMI metric redundant, creates an inconsistency with the multi-indicator approach stated in the abstract, and weakens the overall integrity of the research.

**Response:** Based on your feedback, I agree that this issue has indeed arisen. Consequently, I have incorporated an analysis of the correlation between SSMI and vegetation NPP into my assessment of drought impacts on vegetation NPP. Additionally, I calculated changes in vegetation NPP across different drought severity levels defined by SSMI. By combining analyses of vegetation NPP impacts using both SPEI and SSMI drought indices, I have obtained more scientifically robust results.

**Please see below for the details: “** On a monthly scale, SPEI-1 showed a positive correlation with NPP in 90.93% of cases, with only 8.21% being statistically significant. Meanwhile, SSMI-1 exhibited positive correlations in 85.56% of cases, but only 0.01% were statistically significant. Both findings indicate a relatively limited association between vegetation and drought (Fig 7). At the annual scale, the proportion of positive correlations for SPEI-12 decreased to 80.16%, but the proportion of significant correlations increased to 16.5%, with highly correlated areas concentrated in grasslands. The proportion of positive correlations for SSMI-12 further decreased to 68.23%, while the proportion of significant correlations increased to 30.05%. The spatial pattern resembled that of SPEI-12, indicating that the sensitivity of grassland ecosystems to long-term water accumulation was reflected in both indices. At the seasonal scale, the intensity of positive correlations for SPEI-3 peaked during summer at 0.92, with 27.09% of areas exhibiting statistically significant correlations (higher than the annual scale). SSMI-3 showed positive correlations in 76.02% of areas, with 34.13% being significant, and both indices' high-value zones were distributed across grasslands. Winter exhibited 82.27% negative correlations with SPEI-3 (peak −0.91), while winter SSMI-3 showed only 42.25% negative correlations with low significance. This clearly demonstrates that during cold-limited periods, SPEI—based on evapotranspiration-precipitation balance—provides a more physiologically meaningful representation of vegetation water stress.

Regarding responses across vegetation types (Fig 7), grassland ecosystems exhibited the highest sensitivity and strongest correlations to both drought indices. Cultivated land ecosystems showed moderate sensitivity, while woodland ecosystems demonstrated the weakest response. This reflects differing adaptive strategies and response mechanisms to drought stress across vegetation types. Spring and summer, as the peak growing seasons, represent the most sensitive periods for vegetation responses to drought stress. Both SPEI and SSMI exhibit relatively high correlation strengths and significance levels during these seasons. Winter, however, displays unique correlation patterns, particularly with forest ecosystems generally showing negative correlations during this season.

The overall trends observed in the SPEI-based analysis (Fig 8) align with those from the SSMI-based analysis (S2 Fig). Grassland ecosystems exhibited the highest sensitivity, showing a gradual decline in NPP as drought severity increased. In the SSMI-based analysis, grassland NPP changes demonstrated a positive response to drought, though the magnitude of NPP variation was smaller compared to the SPEI-based analysis. Under various drought levels, NPP changes in cropland ecosystems exhibited trends similar to grasslands, though the decline was less pronounced. Croplands also demonstrated characteristics consistent with human cultivation practices: during drought conditions (excluding extreme drought), both cultivation and early crop growth phases showed positive ΔNPP change rates (0%-30%), indicating a growth phase. Analysis based on SSMI drought levels revealed that ΔNPP in croplands consistently exceeded that in grasslands under drought stress. Cropland NPP exhibited lower sensitivity to soil moisture changes than grasslands, primarily attributed to the moderating effects of agricultural management practices. Forest ecosystems demonstrated distinct NPP variation patterns across drought levels compared to both grasslands and croplands. Results based on SPEI drought levels showed consistent trends with those based on SSMI drought levels, though specific magnitude differences existed. Under mild and moderate drought conditions, NPP changes in forest ecosystems were relatively minor. Only under severe and extreme drought conditions did forest NPP exhibit more pronounced declines, though the magnitude of these declines remained lower than that observed in grassland ecosystems. ” ( Pages 14-16, Lines 279 -325 )

**Comment4：** In section 3.1 of the results and analysis, only the changing trends of SPEI-12 and SSMI are presented graphically. Although the text states that "the trajectories of the two changes are nearly identical," no correlation or significance test results are provided. It is recommended that the authors include a correlation analysis between SPEI and SSMI to quantitatively validate their consistency, thereby enhancing the scientific rigor and persuasiveness of the conclusion.

**Response:** Based on your suggestions, I conducted a correlation analysis and significance test between SPEI and SSMI. The results indicate that the two indices exhibit a positive correlation at a rate of 91.21%, with a significant positive correlation of 54.61%. Therefore, the combination of SPEI and SSMI, as well as the validation of SPEI by SSMI, is scientifically sound.

**Please see below for the details:**

**References:** “ The correlation between SPEI and SSMI is extremely high (the positive correlation reaches 91.21%, and in 54.61% of the regions, this correlation is statistically significant). ” ( Page 11, Lines 211-213 )

**Comment5：**Since the manuscript lists the SSMI and concludes that " grasslands show higher drought correlations than other types due to shallow, dense roots sensitive to surface moisture, with strong event synchrony," it would be beneficial to supplement this with a "comparison of the correlations between NPP and SSMI across different vegetation types (grassland/woodland/farmland)." This addition would enhance the persuasiveness and scientific value of the study.

**Response:** To enhance the scientific rigor of the study, I expanded the correlation analysis between NPP and SSMI to include different vegetation types (grassland, cropland, and woodland). Additionally, I conducted a comparative analysis between the correlations of SPEI with NPP across various vegetation types and the correlations of SSMI with NPP across the same vegetation types.

**Please see below for the details:**

**References: “** Regarding responses across vegetation types (Fig 7), grassland ecosystems exhibited the highest sensitivity and strongest correlations to both drought indices. Cultivated land ecosystems showed moderate sensitivity, while woodland ecosystems demonstrated the weakest response. This reflects differing adaptive strategies and response mechanisms to drought stress across vegetation types. Spring and summer, as the peak growing seasons, represent the most sensitive periods for vegetation responses to drought stress. Both SPEI and SSMI exhibit relatively high correlation strengths and significance levels during these seasons. Winter, however, displays unique correlation patterns, particularly with forest ecosystems generally showing negative correlations during this season. ” ( Page 15, Lines 296-303 )

**Comment6：**The article uses SPEI-1, SPEI-3, and SPEI-12 to represent different drought timescales, but the data processing methods section does not explain what these specific indices represent or how they are calculated.

**Response:** The fundamental concepts of SPEI-1, SPEI-3, and SPEI-12 have been supplemented, and the basic calculation methods have also been briefly described.

**Please see below for the details: “** SPEI-1 is derived from monthly water balance data that is non-cumulative, fitted to a probability distribution, and standardized. It represents the monthly water balance deficit or surplus. This index measures very short-term, near-real-time meteorological drought conditions. It is primarily controlled by monthly precipitation and temperature (which influence evapotranspiration). SPEI-3 is derived from a three-month sliding cumulative monthly water balance, followed by probability distribution fitting and normalization. It represents the cumulative water balance over the past three months (including the current month). It is a seasonal-scale indicator reflecting seasonal moisture trends. SPEI-12 is derived by sliding and accumulating monthly water balances over 12 months, followed by probability distribution fitting and normalization. It represents the cumulative water balance deficit over the past 12 months (including the current month). As an annual-scale indicator, it reflects long-term hydrometeorological trends. ” (Page 7, Lines 133-143 )

**References：**

**Comment7：**The manuscript currently combines results description with mechanistic explanations in Section 3.3.2 ("Analysis of the impact of drought on NPP"). For instance, the statement: " Grasslands show higher drought correlations than other types due to shallow, dense roots sensitive to surface moisture, with strong event synchrony" Such causal explanations belong to ecological mechanism inferences and should be placed in the Discussion section rather than in Results. The Results section should solely report factual findings, while the Discussion should interpret the underlying reasons and connect them to relevant research papers.

**Response:** Based on your feedback, I have removed the ecological mechanism explanation regarding vegetation from the results section and relocated it to the “Discussion” section. Within the discussion, I further analyze the ecological mechanisms underlying this phenomenon by examining the correlation between drought indices (SPEI and SSMI) and NPP, as well as the variation in vegetation NPP across different drought severity levels. This analysis includes comparisons with findings from other studies.

**Please see below for the details:** “Both the correlation between SPEI and grassland NPP and the correlation between SSMI and NPP were higher than those for other vegetation types (Fig 7). Additionally, changes in grassland NPP were the most pronounced across different drought severity levels (Fig 8 and S2). This result aligns with findings by Lyu et al. [66], who demonstrated that grassland vegetation, characterized by shallow and densely distributed roots, exhibits heightened sensitivity to surface water availability. Consequently, grasslands demonstrate stronger synchrony across various drought events compared to other vegetation types. In correlation analyses with drought indices (SPEI and SSMI), forest NPP exhibited lower correlation coefficients than other vegetation types. Under varying drought severity levels, forest NPP showed relatively minor fluctuations, with significant changes occurring only under extreme drought conditions. This further demonstrates that trees possess a degree of resistance to environmental changes and exhibit a certain degree of lag in their response to such changes. This conclusion aligns with the findings of Xiao et al. [67] ” (Pages 19-20, Lines 387-399 )

**References：**Lyu, H., Wang, X., Song, N.P., Chen, J., Zhang, Y.F., Wu, X.D., Yu, D., Yang, X.G., Wang, L., and Chen, L. Response of soil moisture dynamics in four typical herbaceous communities to extreme drought precipitation in the desert steppe. Journal of Soil and Water Conservation, 2023, 37(6): 145-152. https://doi.org/10.13870/j.cnki.stbcxb.2023.06.019.

Xiao, J.Y., Zhang, W.Y., Mu, Y.M., Lyu, L.X. Differences of drought tolerance of the main tree species in Dongling Mountain, Beijing, China as indicated by tree rings. Chin J Appl Ecol. 2021;32:3487–3496. https://doi.org/10.13287/j.1001-9332.202110.001.

**Comment8：**Figure 7 claims that NPP and SPEI show a weak correlation at the monthly scale but a significantly positive correlation at the annual scale. However, the third radar chart in Figure 7—purportedly showing annual-scale correlation coefficients—still displays distinct correlation values for each month (January to December). This suggests that the so-called "annual-scale" analysis was likely performed by correlating monthly NPP values with SPEI-12, rather than using annual aggregated NPP data with SPEI-12. Such an approach is statistically inappropriate, and the authors should provide a clarification on this issue.

**Response:** Thanks to your constructive feedback, I discovered that an inappropriate calculation method had indeed been used. Therefore, I recalculated the SPEI and SSMI drought indices against NPP for different vegetation types across various time scales, yielding the new results presented in Figure 7.

**Please see below for the details:** “ Regarding responses across vegetation types (Fig 7), grassland ecosystems exhibited the highest sensitivity and strongest correlations to both drought indices. Cultivated land ecosystems showed moderate sensitivity, while woodland ecosystems demonstrated the weakest response. This reflects differing adaptive strategies and response mechanisms to drought stress across vegetation types. Spring and summer, as the peak growing seasons, represent the most sensitive periods for vegetation responses to drought stress. Both SPEI and SSMI exhibit relatively high correlation strengths and significance levels during these seasons. Winter, however, displays unique correlation patterns, particularly with forest ecosystems generally showing negative correlations during this season. ” (Page 15, Lines 296-303 )

**Comment9：**In Section 4.2 of the Discussion, the authors state: " Human activities constitute significant drivers of NPP dynamics and necessitate explicit consideration in ecological assessments." However, the entire research framework is confined to meteorological variables and does not incorporate any human activity-related data or analysis, which undermines the logical coherence of the manuscript. It is recommended that the authors reframe this statement as a "future research prospect," clarifying that human activities could be a potential driving factor to be considered in future studies rather than being presented as part of the current.

**Response:** Since this paper primarily investigates the impact of drought on vegetation NPP, it lacks consideration of human activities. Therefore, based on your suggestion, I have framed the study of human activities as a key factor to be addressed in future research.

**Please see below for the details:** “ This study primarily examines the impact of drought on net primary productivity (NPP) in vegetation. Results indicate that drought events significantly suppress vegetation productivity, with varying response levels across different vegetation types. Notably, human activities constitute a critical factor influencing NPP dynamics and must be fully considered in ecological assessments [68]. In the mountainous regions of Shandong, land-use changes accounted for 68.4% of the variation in net primary productivity [69], while the establishment of nature reserves enhanced regional ecosystem services by 80% [70]. However, spring-planted crops remain vulnerable to short-term drought due to their shallow root systems [71]; long-term irrigation effectively mitigates drought stress—demonstrating the spatiotemporal impacts of human activities on vegetation-climate interactions. As this study focuses on natural mechanisms, the quantitative role of human activities remains unexamined. Future research should integrate the coupled effects of natural and anthropogenic factors, using multi-source data fusion and model simulations to discern the relative contributions of different drivers, thereby providing a scientific basis for sustainable ecosystem management. ” ( Page 20, Lines 400-412 )

**References：**Yan, W., Wang, Y., Chaudhary, P., et al. Effects of climate change and human activities on net primary production of wetlands on the Zoige Plateau from 1990 to 2015. Glob Ecol Conserv. 2022;35:e02052. https://doi.org/10.1016/j.gecco.2022.e02052.

Wu, Y.Y., Yang, J., Luo, G., et al. The impact of climate change and human activities on the spatial and temporal variations of vegetation NPP in the hilly-plain region of Shandong Province, China. Forests. 2024;15:512. https://doi.org/10.3390/f15060898.

Liu, Y., Zhao, W.W., Zhang, Z.J., et al. The role of nature reserves in conservation effectiveness of ecosystem services in China. J Environ Manage. 2023;342:118228. https://doi.org/10.1016/j.jenvman.2023.118228.

Kabal, S., Gill, S.K., Jalota, S.K. Previous and current crop effects on early-season root growth and growing season's soil moisture under dryland agriculture in temperate climate. J Agric Sci. 2021;13(5):50-65. https://doi.org/10.5539/jas.v13n5p50.

**Comment10：**The current discussion remains insufficiently in-depth and lacks specificity. It is necessary to build on the research findings presented in the manuscript, supplemented by extensive references to relevant peer-reviewed literature, to deepen the understanding of the climate-sensitive transition zones of North China' uniqueness and develop concrete inferences.

**Response:** During the discussion, the argument that “North China is a highly climate-sensitive transitional zone” was not explored in sufficient depth. Therefore, I will address this from the perspectives of climate and vegetation. North China exhibits both pronounced climatic gradients in temperature and precipitation, as well as transitional vegetation patterns characterized by alternating farmland and grassland. These features collectively demonstrate the region's sensitivity to climate change and its transitional nature.

**Please see below for the details:** “ The North China region is a typical climatic transition zone, where precipitation and temperature exhibit distinct gradients, resulting in pronounced transitional characteristics in its ecosystems and agricultural production methods [46]. The North China Plain also serves as China's most critical grain production base, with its stable grain output holding strategic significance for national food security [47]. The Northern agro-pastoral transition zone is China's largest typical transition zone, with its North China agricultural region serving as a transitional area between agricultural and livestock systems that is highly sensitive to climate change [48-49]. This study reveals that the humid and semi-humid warm-temperate region of North China, as a climate-sensitive transition zone, exhibits rapid responses in net primary productivity to drought events. This finding aligns with previous research highlighting the sensitivity of vegetation-climate interactions in this area [50]. ” ( Page 17, Lines 340-349 )

**References：**

**Comment11：**Figure S1 presents a "validation" that is more of a visual comparison. There are significant mismatches with historical drought records across multiple years, rendering the conclusion of "demonstrating its applicability for drought monitoring" insufficient and unsubstantiated.

**Response:** You have raised questions regarding the validity and reliability of the results in Figure S1. I acknowledge that the analysis for validating SPEI data contained flaws. Therefore, I compared all drought events identified by SPEI (defined as SPEI ≤ -1) from 2001 to 2020 with all drought events documented in the corresponding 20-year period of the Chinese Meteorological Disaster Yearbook. Calculations yielded the Probability of Detection (POD = 0.6957), False Alarm Ratio (FAR = 0.1579), and Critical Success Index (CSI = 0.7273). The results demonstrate the effectiveness of SPEI in identifying droughts.

**Please see below for the details: “** By comparing selected historical drought events with SPEI values from the 2001–2021 China Meteorological Disaster Yearbook, we calculated the hit rate (POD=0.6957), false alarm rate (FAR=0.1579), and critical success index (CSI=0.7273). This confirms that SPEI effectively identifies historical drought events in the humid and semi-humid temperate regions of North China, demonstrating its practicality for drought monitoring (S1 Fig). ” ( Page 11, Lines 207-213 )

**References：**

**Comment12：** Figure S2 suffers from poor readability.

Firstly, the drought severity classifications ("Mild drought" "Moderate drought" and "Severe drought") shown in the figure are not based on standard SPEI threshold criteria. For instance, in the panel labeled "Severe Drought," some SPEI values remain above -0.5, which does not constitute a drought condition according to established classification systems.

Secondly, the comparison is confounded by the fact that the data for different vegetation types correspond to entirely different time periods. Consequently, the observed differences in vegetation response are likely contaminated by interannual climatic variability and other non-drought factors, making it impossible to isolate the specific impact of drought on NPP.

To improve the interpretability of the figure, it is recommended that the response curves for different land cover types be plotted using distinct colors.

**Response:** Based on your feedback, I identified an issue with the SPEI classification in Figure S2. For instance, the impact of a single severe drought event on vegetation NPP cannot be directly discerned from the figure. This is because moderate or mild droughts may occur again after a severe drought, making it impossible to attribute the effect solely to the severe event. Therefore, I adopted drought classification based on drought indices (SPEI and SSMI) to calculate changes in vegetation NPP relative to baseline values (the average vegetation NPP during corresponding drought-free periods) under different drought levels. This approach reflects the impact of drought.

**Please see below for the details:** “ The overall trends observed in the SPEI-based analysis (Fig 8) align with those from the SSMI-based analysis (S2 Fig). Grassland ecosystems exhibited the highest sensitivity, showing a gradual decline in NPP as drought severity increased. In the SSMI-based analysis, grassland NPP changes demonstrated a positive response to drought, though the magnitude of NPP variation was smaller compared to the SPEI-based analysis. Under various drought levels, NPP changes in cropland ecosystems exhibited trends similar to grasslands, though the decline was less pronounced. Croplands also demonstrated characteristics consistent with human cultivation practices: during drought conditions (excluding extreme drought), both cultivation and early crop growth phases showed positive ΔNPP change rates (0%-30%), indicating a growth phase. Analysis based on SSMI drought levels revealed that ΔNPP in croplands consistently exceeded that in grasslands under drought stress. Cropland NPP exhibited lower sensitivity to soil moisture changes than grasslands, primarily attributed to the moderating effects of agricultural management practices. Forest ecosystems demonstrated distinct NPP variation patterns across drought levels compared to both grasslands and croplands. Results based on SPEI drought levels showed consistent trends with those based on SSMI drought levels, though specific magnitude differences existed. Under mild and moderate drought conditions, NPP changes in forest ecosystems were relatively minor. Only under severe and extreme drought conditions did forest NPP exhibit more pronounced declines, though the magnitude of these declines remained lower than that observed in grassland ecosystems. ” ( Pages 15-16, Lines 308-325 )

**References：**

**Comment13：**The presentation of data sources in the manuscript is inconsistent. It is recommended to standardize the formatting of data citations throughout the article, avoiding non-standard forms such as "from [26-27]".

**Response:** The citation format for data sources has been revised, and the annotation format for data sources has been standardized.

**Please see below for the details:** “ This study utilized meteorological data (average temperature [25] and monthly precipitation[26] for China, 1901-2023), and an aridity index (AI) dataset (1 km resolution, China, 1901-2024) from Peng [27]. ” (Page 6, Lines 111-113 )

**Comment14：**The manuscript contains numerous issues with its figures. The resolution of all images is too low, making text and details difficult to distinguish.

In Figure 1, the scale bars are inconsistently labeled as "kilometers" and "Kilometers".

Figures 2 and 3 have incomplete borders in their legends.

Figure 3 is confusing in its layout. The four spatial distribution maps in the middle, marked with years, are actually expanded from subplot a. However, their current arrangement alongside the main figure creates an unclear information hierarchy, making it difficult to recognize them as elaborations of subplot a. The authors should reconsider the overall layout design.

Figure 4 lacks a title for its legend, and "spei" should be written in its standard form as "SPEI".

Subplots c and d in Figure 5 should each include a north arrow and a scale bar.

Figure 6 lacks both a north arrow and a scale bar. Furthermore, while the caption describes it as showing "correlation coefficients," the figure itself only visually represents correlations without displaying the actual numerical values.

**Response:** Thank you for your thoughtful feedback. I have revised each image according to your suggestions.

**Reviewer#2：**

**Response:** Thanks for the positive comments.

**Major concerns:**

**Comment1：**Extreme droughts have a significant impact on ecosystems. The paper defines drought events of different degrees. It is suggested that future research could further analyze the impact of major drought events and their frequency during the growing season and even the early growing season on NPP.

**Response:** Your feedback has been highly constructive for my paper and will be a key focus in my subsequent work. I calculated monthly changes in vegetation NPP across different drought severity levels from 2001 to 2020, allowing for a straightforward illustration of how monthly vegetation NPP was impacted during severe or extreme drought events. Reviewing the literature reveals no clearly defined boundaries for the growing season in North China. However, most studies mention a growing season spanning late March to early April and late October to early November. Therefore, I can only briefly explore the impact of drought events on vegetation NPP during the early growing season based on monthly changes in vegetation NPP under different drought levels. Future research will delve deeper into the relationship between drought and seasonal vegetation NPP, more precisely define the growth season range in North China, and investigate how drought events affect vegetation NPP during different phases of the growing season.

**Please see below for the details:** “ Under the four drought severity levels classified by SPEI (Fig 8), ΔNPP exhibited negative anomalies across grasslands, croplands, and woodlands during March–May (early growing season), with the magnitude of negative anomalies increasing as drought severity intensified. Within the same severity level, grasslands showed the deepest negative anomalies, while woodlands exhibited the shallowest. From June to August (the peak growing season), the magnitude of negative anomalies for the same vegetation types exceeded that observed from March to May. Results based on SSMI-classified drought levels also showed the same trend (S2 Fig). This indicates that drought during the early growing season has a persistent weakening effect on NPP in later stages. ” (Page 16, Lines 330-337 )

**References：**

**Comment2：**The authors still use SPEI when evaluating the relationship between environmental factors and farmland. Why not use SSMI? Please note that irrigation has a significant impact on mitigating major droughts in farmland. It is recommended that the authors re-analyze using SSMI, even if the results are not significantly different. This insignificant difference may be due to low resolution (1 km), which makes it impossible to identify soil moisture changes caused by irrigation.

**Response:** In previous drafts, the exclusive use of SPEI as an indicator to assess drought's impact on vegetation NPP did indeed reveal shortcomings. Based on your feedback, we have supplemented the SPEI analysis examining drought's relationship with NPP across different vegetation types (grasslands, croplands, and woodlands) by incorporating SSMI. This includes analyzing the correlation between SSMI and NPP for various vegetation types, as well as documenting changes in vegetation NPP under different drought severity levels.

**Please see below for the details:** “ Regarding responses across vegetation types (Fig 7), grassland ecosystems exhibited the highest sensitivity and strongest correlations to both drought indices. Cultivated land ecosystems showed moderate sensitivity, while woodland ecosystems demonstrated the weakest response. This reflects differing adaptive strategies and response mechanisms to drought stress across vegetation types. Spring and summer, as the peak growing seasons, represent the most sensitive periods for vegetation responses to drought stress. Both SPEI and SSMI exhibit relatively high correlation strengths and significance levels during these seasons. Winter, however, displays unique correlation patterns, particularly with forest ecosystems generally showing negative correlations during this season. ” ( Page 15, Lines 296-303 )

“ Regarding responses across vegetation types (Fig 7), grassland ecosystems exhibited the highest sensitivity and strongest correlations to both drought indices. Cultivated land ecosystems showed moderate sensitivity, while woodland ecosystems demonstrated the weakest response. This reflects differing adaptive strategies and response mechanisms to drought stress across vegetation types. Spring and summer, as the peak growing seasons, represent the most sensitive periods for vegetation responses to drought stress. Both SPEI and SSMI exhibit relatively high correlation strengths and significance levels during these seasons. Winter, however, displays unique correlation patterns, particularly with forest ecosystems generally showing negative correlations during this season. ” (Pages 15-16, Lines308-325 )

**Minors：**

**Comment1：** What is Figd in Line 314?

**Response:** Thank you for your thorough feedback. This was indeed a careless spelling error on my part, and I have now carefully proofread and corrected it.

**Please see below for the details:** “ This positive effect concentrates in high-warming areas like Shandong Province (0.036℃/a)(Table 3), where most zones show significant NPP increases (Fig. 5d). ” ( Page 18, Lines 377-379 )

**Comment2：**Please unify the caption format for figures and tables in the document.

**Response:** Based on your feedback, the titles of all figures and tables in the manuscript have been standardized.

**Comment3：**Fig. 5: The CASA simulation results and MODIS NPP product results are good, but the sample size is small, and it is unclear whether all land cover types are covered or their distribution. The authors are requested to provide a detailed description of the validation design and increase the validation sample size.

**Response:** The validation of the CASA model's simulation results against MODIS satellite NPP data was determined using the Coefficient of Determination (R^2^). R^2^ essentially measures how well a regression model fits the data, specifically the proportion of variation in the dependent variable (target variable) that the model can explain. Therefore, R^2^ is used to assess the reliability of the simulated values. Based on your feedback, considering the validation sample size was indeed somewhat small and did not cover all land cover types, I increased the number of validation samples. I extracted the annual averages for different land cover types (grassland, cropland, forest, water bodies, built-up areas, and snow/ice bare land) and calculated the R^2^ between simulated and observed values. This yielded a satisfactory result of R^2^ = 0.8766, which passed the significance test.

**Please see below for the details:** “ However, based on the annual averages of different land use types in the study area, the validation of MOD17A3 data (Fig 5a) confirms that the outputs generated by CASA are consistent with the baseline data (R²=0.8766, p<0.01), thus supporting the reliability of these regional estimates. ” ( Page 12, Lines 243-246 )

**Figure:**

**Comment1：**Fig. 6: The font size for the correlation data is too small; please redraw it.

**Response:** Based on your feedback, the font size in the diagram has been modified and adjusted.

**Comment2：**Fig. S2: The authors listed drought conditions for three land cover types under different drought levels. To better support the authors' conclusions in L262-273 of the paper, it is suggested to use an overlay analysis method, overlaying major, moderate, and mild drought events within the study period. The results of this analysis will be more valuable.

**Response:** Thank you for your feedback. I have acknowledged the shortcomings in Figure S2. I have categorized drought levels using different indices (SPEI and SSMI) and conducted an overlay analysis of drought conditions across these levels with NPP data from various vegetation types. This approach reveals how vegetation NPP varies under different drought intensities, enabling a more scientifically rigorous analysis of drought impacts on vegetation NPP.

**Please see below for the details:** “ The overall trends observed in the SPEI-based analysis (Fig 8) align with those from the SSMI-based analysis (S2 Fig). Grassland ecosystems exhibited the highest sensitivity, showing a gradual decline in NPP as drought severity increased. In the SSMI-based analysis, grassland NPP changes demonstrated a positive response to drought, though the magnitude of NPP variation was smaller compared to the SPEI-based analysis. Under various drought levels, NPP changes in cropland ecosystems exhibited trends similar to grasslands, though the decline was less pronounced. Croplands also demonstrated characteristics consistent with human cultivation practices: during drought conditions (excluding extreme drought), both cultivation and early crop growth phases showed positive ΔNPP change rates (0%-30%), indicating a growth phase. Analysis based on SSMI drought levels revealed that ΔNPP in croplands consistently exceeded that in grasslands under drought stress. Cropland NPP exhibited lower sensitivity to soil moisture changes than grasslands, primarily attributed to the moderating effects of agricultural management practices. Forest ecosystems demonstrated distinct NPP variation patterns across drought levels compared to both grasslands and croplands. Results based on SPEI drought levels showed consistent trends with those based on SSMI drought levels, though specific magnitude differences existed. Under mild and moderate drought conditions, NPP changes in forest ecosystems were relatively minor. Only under severe and extreme drought conditions did forest NPP exhibit more pronounced declines, though the magnitude of these declines remained lower than that observed in grassland ecosystems. ” ( Pages 15-16, Lines 308-325 )
