# Supplementary material for: Drought characteristics and their impact on vegetation net primary productivity in the climate-sensitive transition zones of North China
Source: PLoS One. 2026 Feb 25;21(2):e0343746. doi: 10.1371/journal.pone.0343746 (PMC12935246; doi:10.1371/journal.pone.0343746)
Supplement: Attachment — Submitted filename: Response_to_Reviewers_auresp_2.docx [file pone.0343746.s006.docx]

**Response to the Reviewer's Comments**

**Submission ID:** PONE-D-25-53876

**Title:** Drought Characteristics and Their Impact on Vegetation Net Primary Productivity in the Climate-Sensitive Transition Zones of North China

**Comments from the Editor:**

**Response:** We extend our sincere gratitude once again to all editors and reviewers for their valuable comments and suggestions. After carefully considering each piece of feedback, we have made corresponding revisions and improvements to the manuscript to further enhance the clarity and readability of the text.

**Reviewer#1：**

Response: Thanks for the positive comments.

**Comment1**：In the Discussion section 4.1, the manuscript states:"This study uncovers climate-drought coupling in North China's humid and semi-humid warm temperate regions: increased precipitation dominates water balance improvement and meteorological drought reduction. Crucially, precipitation rise offsets temperature-driven evapotranspiration losses, slightly mitigating drought." However, the primary evidence currently provided in the paper is based on correlation analysis. Correlation only indicates a statistical association between variables and cannot directly support mechanistic conclusions such as "offset."

**Response**: Based on your feedback, I have come to fully recognize that certain expressions in the “ Discussion ” section were inappropriate. My original intent was to analyze the causes behind the trend changes in climatic factors and drought conditions within this study during the discussion phase, but the wording fell short. I have revised the text accordingly, now presenting it in three distinct sections: trends in temperature and precipitation changes, overall trends in drought conditions, and an analysis of the causes.

**Please see below for the details**: “ This study also reveals that both temperature and precipitation are showing an upward trend in the humid and semi-humid warm temperate regions of North China. This finding aligns with the results of Zhao et al. [52], which explicitly indicated a significant increase in precipitation after 2012, occurring concurrently with rising temperatures (warming and moistening of the atmosphere). The study area exhibits a slight decrease in the area affected by drought at both the annual and seasonal scales (except for winter), indicating an overall trend of slight drought alleviation. This phenomenon may be related to the findings of Zhou et al. [53], which suggest that increased precipitation mitigates water limitations exacerbated by vegetation recovery and climate warming, thereby slightly alleviating drought conditions. ”( Page 17, Lines 351 - 359 )

**References**：

Zhao, J.C., Li, Q.Q., Ding, Y.H., Liu, Y.Y., Tan, G.R., Shen, X.Y., Wu, Q.Y. 2023. Interdecadal increase of summer precipitation in North China in the early 2010s and its association with atmospheric circulation anomalies. Acta Meteorologica Sinica, 81(5):764–775. https://doi.org/10.11676/qxxb2023.20220198

Zhou, J.L., Liu, Q., Liang, L.Q., Yan, D.H., Yang, Y.T., Wang, X., Sun, T., Li, S.Z., Gan, L.Y., Wu, J.F. 2024. Water constraints enhanced by revegetation while alleviated by increased precipitation on China’s water-dominated Loess Plateau. Journal of Hydrology, 640:131731. https://doi.org/10.1016/j.jhydrol.2024.131731.

**Comment2**：The discussion section needs to further strengthen the differences and synergistic advantages between SPEI and SSMI. It is recommended to integrate the article’s findings on the correlations among SPEI, SSMI, and NPP to clarify the differences between the two indices in drought monitoring and NPP impact assessment, thereby justifying the necessity and advantages of the dual-indicator approach.

**Response**: Thank you for your constructive suggestions. I believe it is very necessary to add further discussions on the SPEI and SSMI indices in the discussion section. I have included discussions on SPEI and SSMI in Section 4.3, covering three aspects: the main differences and respective advantages of SPEI and SSMI, the advantages of comparing them together, as well as relevant literature support. Additionally, the study compares how different vegetation types’ NPP responds to drought (as measured by SPEI or SSMI). In conclusion, using both SPEI and SSMI indicators in conjunction will make the results more scientifically sound.

**Please see below for the details**: “This study primarily employed SPEI and SSMI as indices to characterize drought impacts on vegetation NPP. First, SPEI, as a meteorological drought index, primarily reflects the balance between atmospheric water supply (precipitation) and demand (potential evapotranspiration) [66]. It captures anomalies in atmospheric moisture conditions, serving as an “early warning signal” for vegetation experiencing water stress. Second, SSMI directly reflects actual available soil moisture content, serving as an indicator of water conditions directly accessible to plant roots. It more directly links vegetation physiological processes and productivity [67]. Despite differences between SPEI and SSMI, drought typically begins as meteorological drought, gradually evolves into soil drought, and ultimately impacts vegetation. Combining both indices enables a more comprehensive and accurate revelation of the complex mechanisms by which drought affects vegetation NPP, demonstrating clear synergistic advantages. Afshar et al. in studying the impact of concurrent droughts on global NPP losses, found that significant NPP declines were often accompanied by simultaneous reductions in both SPEI and SSMI, underscoring the critical importance of integrated monitoring of atmospheric and soil drought for assessing ecological risks[68]. This study also demonstrates the synergistic advantages of SPEI and SSMI in more effectively analyzing net primary productivity responses to drought across different vegetation types. Both the correlation between SPEI and grassland NPP and the correlation between SSMI and NPP were higher than those for other vegetation types (Fig 7). Additionally, changes in grassland NPP were the most pronounced across different drought severity levels (Fig 8 and S2). This result aligns with findings by Lyu et al. [69], who demonstrated that grassland vegetation, characterized by shallow and densely distributed roots, exhibits heightened sensitivity to surface water availability. Consequently, grasslands demonstrate stronger synchrony across various drought events compared to other vegetation types. In correlation analyses with drought indices (SPEI and SSMI), forest NPP exhibited lower correlation coefficients than other vegetation types. Under varying drought severity levels, forest NPP showed relatively minor fluctuations, with significant changes occurring only under extreme drought conditions. This further demonstrates that trees possess a degree of resistance to environmental changes and exhibit a certain degree of lag in their response to such changes. This conclusion aligns with the findings of Xiao et al. [70]. This study demonstrates that by comparing SPEI and SSMI with NPP across specific vegetation types, more reliable assessment results can be obtained. ” ( Pages 19 - 20, Lines 390 - 418 )

**References**：

Stagge, J.H., Tallaksen, L.M., Xu, C.Y., et al. 2014. Standardized precipitation-evapotranspiration index (SPEI): Sensitivity to potential evapotranspiration model and parameters. In Servat, E., Paturel, J.-E., Dezetter, A., et al. (Eds.), Hydrology in a Changing World: Environmental and Human Dimensions - Proceedings of FRIEND-Water 2014, 363:367–373. Copernicus Publications. https://doi.org/10.5194/piahs-363-367-2014.

Xie, L.L., Li, Y., Zhang, Z.Y., et al. 2025. Exploring the combined effects of drought and drought-flood abrupt alternation on vegetation using interpretable machine learning model and r-vine copula function. Agricultural and Forest Meteorology, 370:110568. https://doi.org/10.1016/j.agrformet.2025.110568.

Afshar, M.H., Bulut, B., Duzenli, E., et al. 2022. Global spatiotemporal consistency between meteorological and soil moisture drought indices. Agricultural and Forest Meteorology, 316:108848. https://doi.org/10.1016/j.agrformet.2022.108848.

Lyu, H., Wang, X., Song, N.P., Chen, J., Zhang, Y.F., Wu, X.D., Yu, D., Yang, X.G., Wang, L., and Chen, L. Response of soil moisture dynamics in four typical herbaceous communities to extreme drought precipitation in the desert steppe. Journal of Soil and Water Conservation, 2023, 37(6): 145-152. https://doi.org/10.13870/j.cnki.stbcxb.2023.06.019.

Xiao, J.Y., Zhang, W.Y., Mu, Y.M., Lyu, L.X. Differences of drought tolerance of the main tree species in Dongling Mountain, Beijing, China as indicated by tree rings. Chin J Appl Ecol. 2021;32:3487–3496. https://doi.org/10.13287/j.1001-9332.202110.001.

**Comment3：**In Figure 1, the spacing between the scale markers 80 and 160 is too small.

**Response**: Thank you for your detailed suggestions. I have adjusted the scale in Figure 1.
